# Serum uric acid and TG/HDL-C ratio in non-obese working women: an exploratory cross-sectional study

**DOI:** 10.1186/s12902-026-02380-1

**Published:** 2026-06-27

**Authors:** Takasuke Asakawa

**Affiliations:** 1 Setagaya Occupational Health Office, 1-3-8 Setagaya, Setagaya-ku, Tokyo Japan; 2 Asakawa Clinic, Setagaya-ku, Tokyo Japan

**Keywords:** Uric acid, TG/HDL-C ratio, Non-obese women, Cardiometabolic risk, Occupational health

## Abstract

**Background:**

Serum uric acid has been suggested to be associated with metabolic abnormalities; however, its independent role remains unclear, particularly among non-obese individuals.

**Methods:**

We conducted a cross-sectional analysis of 630 non-obese women (body mass index < 25 kg/m^2^) selected from an initial cohort of 892 participants. Individuals receiving lipid-lowering therapy and those with missing data on serum uric acid, triglycerides, or HDL-C were excluded. Blood samples included in this analysis were limited to fasting samples obtained as part of routine occupational health examinations. The TG/HDL-C ratio was used as a surrogate marker related to insulin resistance and cardiometabolic risk, rather than as a direct measure of insulin resistance or clinical cardiovascular outcomes. Associations between serum uric acid and the TG/HDL-C ratio were examined using quartile analysis and multivariable linear regression adjusting for age, BMI, systolic blood pressure, medication use, smoking, alcohol consumption, and weight gain since early adulthood.

**Results:**

The TG/HDL-C ratio increased across quartiles of serum uric acid, particularly in the upper quartiles. In the crude model, serum uric acid was positively associated with the TG/HDL-C ratio (β = 0.060, *p* = 0.010), but this association was attenuated after multivariable adjustment and was no longer statistically significant (β = 0.025, *p* = 0.289). In contrast, BMI (β = 0.063, *p* < 0.001) and weight gain since early adulthood (β = 0.261, *p* < 0.001) showed stronger adjusted associations with the TG/HDL-C ratio. Exploratory subgroup analyses were interpreted cautiously because of limited sample size.

**Conclusions:**

In this exploratory cross-sectional study, serum uric acid was not independently associated with the TG/HDL-C ratio after adjustment for confounding factors. These findings suggest that serum uric acid may reflect underlying metabolic status in non-obese working women; however, this interpretation should be considered hypothesis-generating because of the cross-sectional design and the use of the TG/HDL-C ratio as a surrogate marker.

**Clinical trial number:**

Not applicable.

**Supplementary information:**

The online version contains supplementary material available at https://doi.org/10.1186/s12902-026-02380-1.

## Introduction

Serum uric acid has been widely discussed as a potential marker of metabolic abnormalities, including insulin resistance and dyslipidemia [1, 2]. Elevated uric acid levels have been associated with adverse cardiometabolic profiles, such as increased triglycerides and reduced high-density lipoprotein cholesterol (HDL-C), in both general and occupational populations [3, 4].

The triglyceride-to-HDL-C (TG/HDL-C) ratio is a clinically simple surrogate marker that has been associated with insulin resistance and cardiometabolic risk [5, 6]. However, it should be interpreted as an indirect marker and not as a direct measure of insulin resistance, metabolic disease, or clinical cardiovascular outcomes.

The independent role of uric acid remains controversial. Several studies have reported positive associations between uric acid and metabolic abnormalities [7, 8], but these associations are often attenuated after adjustment for adiposity and lifestyle-related factors. Experimental studies have suggested that uric acid may influence metabolic pathways through mechanisms such as oxidative stress and endothelial dysfunction, whereas epidemiological associations are strongly influenced by obesity, visceral adiposity, and lifestyle.

Most previous studies have focused on general or obese populations. In contrast, the clinical significance of uric acid in non-obese individuals remains unclear despite the growing recognition of “metabolically unhealthy normal-weight” individuals [9]. Non-obese individuals are often regarded as metabolically low-risk, but some may still have early metabolic abnormalities that are not evident from BMI alone.

Furthermore, weight gain since early adulthood has been shown to be associated with metabolic abnormalities, even among individuals with a normal body mass index [10, 11]. Whether the relationship between serum uric acid and cardiometabolic risk markers differs according to long-term weight change has not been fully elucidated.

Therefore, the purpose of the present study was not to establish a direct causal role of uric acid in lipid metabolism, but to explore whether serum uric acid may reflect metabolic status, as assessed by the TG/HDL-C ratio, among non-obese working women. We also examined the influence of weight gain since early adulthood, given its relevance to occupational health examinations and early risk identification.

## Methods

### Study design and population

All female employees aged 20 years or older who underwent routine annual occupational health examinations were eligible for inclusion. This study used fully anonymized secondary data derived from routine occupational health practice; therefore, no study-specific recruitment or refusals occurred. Each participant contributed one observation from a single examination.

A total of 892 women comprised the source population. Participants receiving lipid-lowering therapy a priori were excluded because such treatment directly influences lipid profiles. Individuals with missing data on serum uric acid, triglycerides, or HDL-C were further excluded, resulting in 740 participants. The analysis was restricted to non-obese individuals (body mass index [BMI] <25 kg/m^2^), yielding a final analytical sample of 630 participants.

The exclusions were primarily due to differences in laboratory testing and examination items available across routine occupational health examination programs, rather than clinical selection.

### Ethical considerations

Health examinations were conducted under the Industrial Safety and Health Act of Japan. This study used fully anonymized secondary data derived from routine occupational health practice.

The study protocol was reviewed by the Research Ethics Oversight Committee of Asakawa Clinic, which determined that formal ethical approval was not required and waived the requirement for individual informed consent because no personally identifiable information was accessible to the investigators. A minor protocol amendment submitted to the Hospital Joint Ethics Review Committee underwent committee review and was approved on May 15, 2026 (Approval No. 14000050.20260417-27S388). The approved amendment did not affect the current analysis or study conclusions.

All data were irreversibly anonymized prior to analysis. The study was conducted in accordance with the Ethical Guidelines for Medical and Health Research Involving Human Subjects issued by the Ministry of Health, Labour and Welfare of Japan (2021) and the Declaration of Helsinki.

### Measurements, exposure, and outcome

Health examinations were performed using standardized procedures. Blood samples included in this analysis were limited to fasting samples obtained as part of routine occupational health examinations. Serum uric acid, triglycerides, and HDL-C were measured in certified laboratories. Blood pressure was measured in the seated position after a period of rest. BMI was calculated as weight in kilograms divided by height in meters squared.

Serum uric acid was analyzed both as a continuous variable and by quartiles defined within the study population (Q1 ≤ 3.7 mg/dL, Q2 3.8–4.2 mg/dL, Q3 4.3–4.9 mg/dL, Q4 ≥ 5.0 mg/dL).

The primary outcome was the triglyceride-to-HDL cholesterol ratio (TG/HDL-C ratio), used as a surrogate marker associated with insulin resistance and cardiometabolic risk. The TG/HDL-C ratio was not interpreted as a direct clinical outcome.

Covariates included age, BMI, systolic blood pressure, use of antihypertensive medication, use of antidiabetic medication, current smoking status (yes/no), alcohol consumption (yes/no), and weight gain since early adulthood (≥10 kg vs. <10 kg). Weight gain since early adulthood was assessed using questionnaire data obtained as part of routine health checkups.

Information on lifestyle factors and medical history was obtained from standardized questionnaire data provided as part of routine health checkups. These variables were collected as binary (yes/no) responses based on questionnaires administered at each facility. As the original questionnaires varied across facilities, a harmonized dataset was used for analysis (Supplementary Table S1).

### Statistical analysis

Continuous variables are presented as mean ± standard deviation (SD), and categorical variables as counts and percentages.

Differences across serum uric acid quartiles were assessed using one-way analysis of variance (ANOVA) for continuous variables and the chi-square test for categorical variables. Linear trends across quartiles were evaluated by assigning ordinal values to each quartile and including them as continuous variables in regression models.

Multivariable linear regression analyses were performed to examine the association between serum uric acid and the TG/HDL-C ratio. Covariates were selected a priori based on clinical relevance and previous literature.

All statistical analyses were performed using Microsoft Excel version 2021 (Microsoft Corporation) with the Data Analysis ToolPak, employing ordinary least squares regression. Regression coefficients and 95% confidence intervals (CIs) were calculated using the Student’s t-distribution with n-k-1 degrees of freedom (where *n* = 630 and k = 9 independent variables). Variance inflation factors (VIF) were assessed to evaluate multicollinearity, with all VIF values < 5 indicating no major multicollinearity concerns. Residuals were visually inspected to confirm model assumptions including normality and homogeneity of variance. A two-sided *p*-value < 0.05 was considered statistically significant.

## Results

The baseline characteristics of the study population according to serum uric acid quartiles are shown in Table 1.

**Table 1:** Baseline characteristics of the study population according to quartiles of serum uric acid

Variables	Q1 ≤ 3.7 (*n* = 170)	Q2 3.8–4.2 (*n* = 151)	Q3 4.3–4.9 (*n* = 167)	Q4 ≥ 5.0 (*n* = 142)	*p* value
Age (years)	39.9 ± 9.7	40.0 ± 10.3	41.4 ± 11.2	41.8 ± 11.7	0.264
BMI	20.0 ± 2.1	20.1 ± 2.0	20.7 ± 2.2	21.1 ± 2.3	<0.001
SBP (mmHg)	108 ± 13.5	110 ± 13.5	112 ± 14.5	112 ± 14.4	0.025
TG/HDL-C	0.90 ± 0.41	0.91 ± 0.47	0.99 ± 0.60	1.03 ± 0.60	0.058
Hypertension medication	2 (1.2%)	4 (2.6%)	9 (5.4%)	11 (7.7%)	0.019
Diabetes medication	1 (0.6%)	0 (0%)	0 (0%)	2 (1.4%)	0.244
Current smoker	0 (0%)	4 (2.6%)	5 (3.0%)	8 (5.6%)	0.024
Alcohol use	89 (52.4%)	88 (58.3%)	111 (66.5%)	96 (67.6%)	0.015
Weight gain ≥ 10 kg	14 (8.2%)	6 (4.0%)	26 (15.6%)	22 (15.5%)	0.001

Values are presented as mean ± standard deviation or number (%). Serum uric acid quartiles are shown in mg/dL. *p* values were calculated using one-way analysis of variance for continuous variables and the chi-square test for categorical variables. TG/HDL-C, triglyceride-to-high-density lipoprotein cholesterol ratio; SBP, systolic blood pressure

**Fig. 1:**
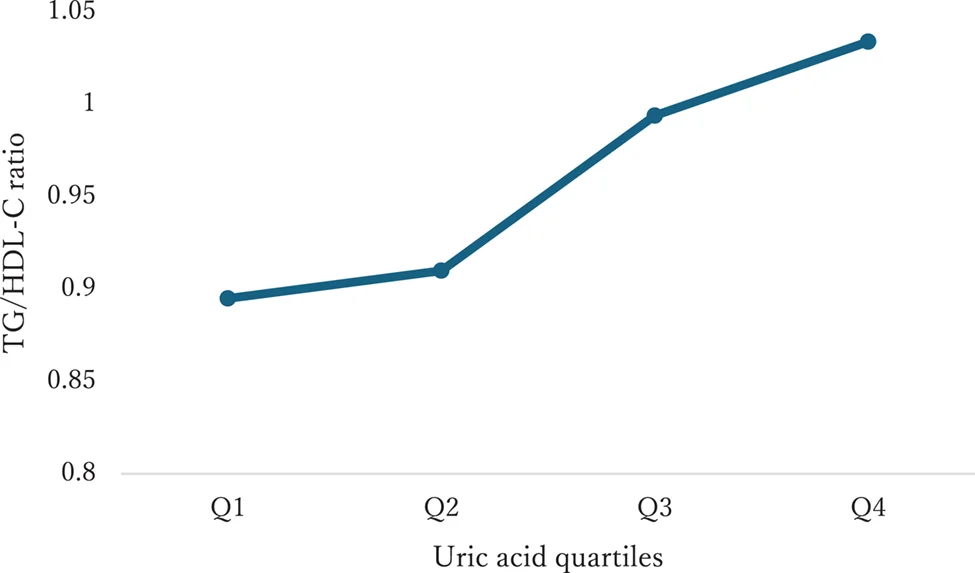
Mean TG/HDL-C ratio (± SD) across quartiles of serum uric acid in non-obese working women. Q1 (*n* = 170), Q2 (*n* = 151), Q3 (*n* = 167), Q4 (*n* = 142). p for trend = 0.032 (unadjusted). Error bars indicate standard deviation (SD)

The TG/HDL-C ratio increased across quartiles of serum uric acid, with higher values observed in the upper quartiles (Fig. 1). The mean TG/HDL-C ratios were 0.90, 0.91, 0.99, and 1.03 from the lowest to the highest uric acid quartiles, indicating an overall upward trend.

In the crude model, serum uric acid was positively associated with the TG/HDL-C ratio (β = 0.060, 95% CI 0.014 to 0.106, *p* = 0.010; R^2^ = 0.010). However, this association was attenuated and was no longer statistically significant after multivariable adjustment.

In multivariable linear regression analysis (Table 2), serum uric acid was not independently associated with the TG/HDL-C ratio (β = 0.025, 95% CI −0.021 to 0.071, *p* = 0.289). In contrast, BMI (β = 0.063, 95% CI 0.043 to 0.082, *p* < 0.001) and weight gain since early adulthood (β = 0.261, 95% CI 0.124 to 0.397, *p* < 0.001) showed stronger adjusted associations with the TG/HDL-C ratio. Systolic blood pressure showed an inverse borderline association (β = −0.003, 95% CI −0.006 to 0.000, *p* = 0.049). The overall model showed limited explanatory power (R^2^ = 0.128, adjusted R^2^ = 0.115).

**Table 2:** Multivariable linear regression analysis of factors associated with the TG/HDL-C ratio

Variables	β (95% CI)	p value
Uric acid	0.025 (−0.021 to 0.071)	0.289
Age	0.001 (−0.003 to 0.004)	0.742
BMI	0.063 (0.043 to 0.082)	<0.001
SBP	−0.003 (−0.006 to 0.000)	0.049
Hypertension medication	−0.094 (−0.302 to 0.114)	0.375
Diabetes medication	0.201 (−0.364 to 0.766)	0.485
Current smoker	0.082 (−0.162 to 0.326)	0.511
Alcohol use	−0.077 (−0.157 to 0.004)	0.061
Weight gain ≥ 10 kg	0.261 (0.124 to 0.397)	<0.001

β, regression coefficient; CI, confidence interval; BMI, body mass index; SBP, systolic blood pressure. The model was adjusted for age, BMI, systolic blood pressure, medication use, smoking, alcohol consumption, and weight gain since early adulthood

Exploratory subgroup analyses were performed according to weight gain status. Because the number of participants was limited, particularly in the subgroup with weight gain ≥ 10 kg, these findings should be interpreted cautiously and regarded as hypothesis-generating. Serum uric acid was not clearly associated with the TG/HDL-C ratio in either subgroup (Supplementary Table S2).

## Discussion

In this exploratory cross-sectional study of non-obese working women, the TG/HDL-C ratio increased across quartiles of serum uric acid; however, this association was not independent after adjustment for confounding factors. These exploratory findings suggest that serum uric acid may reflect underlying metabolic status rather than function as an independent determinant of cardiometabolic risk as assessed by the TG/HDL-C ratio. Given the cross-sectional design, these results should be interpreted cautiously and should not be considered evidence of a causal relationship.

The rationale for examining this association was to clarify how serum uric acid should be interpreted in non-obese individuals, who are often considered metabolically low-risk but may still have early metabolic abnormalities. The present study did not aim to establish a direct causal effect of uric acid on lipid metabolism. Rather, it explored whether uric acid is associated with a simple surrogate marker of insulin resistance and cardiometabolic risk in an occupational health examination setting.

Previous studies have consistently reported that elevated serum uric acid is associated with insulin resistance and dyslipidemia [1, 2]. In addition, several epidemiological studies have demonstrated that higher uric acid levels are linked to the development of metabolic syndrome and hypertension [12–14]. Experimental studies have also suggested that uric acid may influence metabolic pathways through mechanisms such as oxidative stress and endothelial dysfunction [15]. In particular, uric acid has been linked to adverse lipid profiles, including higher triglycerides and lower HDL-C levels [3, 4]. Consistent with these findings, we observed an increase in the TG/HDL-C ratio across uric acid quartiles.

However, the independent role of uric acid remains controversial. Several studies have demonstrated that the association between uric acid and metabolic risk markers is attenuated after adjustment for adiposity and related factors [7]. Similar findings have been reported in Japanese populations, where the association between serum uric acid and metabolic syndrome was substantially influenced by metabolic factors such as obesity and lifestyle [16]. Our findings are consistent with these reports, as the association between uric acid and the TG/HDL-C ratio was not statistically significant after multivariable adjustment. This attenuation suggests that the crude association may be partly explained by adiposity, weight gain, and related lifestyle factors. In addition, the modest explanatory power of the multivariable model supports an exploratory interpretation of the present findings, indicating that factors not captured in this analysis may also contribute to variation in the TG/HDL-C ratio.

In our study, BMI and weight gain since early adulthood showed stronger adjusted associations with the TG/HDL-C ratio than serum uric acid. This wording is intended to describe the observed statistical associations and not to imply causal or more direct biological effects, which cannot be determined from the cross-sectional design. These findings are consistent with previous evidence showing that long-term weight gain is associated with metabolic abnormalities, even among individuals with normal BMI [10, 11].

The inverse association between systolic blood pressure and the TG/HDL-C ratio observed in the multivariable model was unexpected and only borderline significant. Although multicollinearity was not a major concern, this estimate may reflect residual confounding or model instability. Therefore, this finding should be interpreted cautiously and should not be regarded as evidence of a clinically meaningful inverse relationship.

The subgroup analyses according to weight gain status should be regarded as hypothesis-generating. Although the patterns appeared to differ by weight gain status, the number of participants with weight gain ≥ 10 kg was limited, and serum uric acid was not clearly associated with the TG/HDL-C ratio in either subgroup. Therefore, these subgroup findings should not be overinterpreted and require confirmation in larger studies.

These findings may be relevant in the context of “metabolically unhealthy normal-weight” individuals [9]. Even among non-obese populations, metabolic abnormalities can develop, and serum uric acid may provide contextual information regarding metabolic status. However, the present findings do not support serum uric acid as an independent determinant of cardiometabolic risk, and they should not be used to infer clinical outcomes.

## Limitations

This study has several limitations. First, due to its cross-sectional design, causality cannot be established. Second, the TG/HDL-C ratio is a surrogate marker related to insulin resistance and cardiometabolic risk and should not be interpreted as a direct measure of insulin resistance or clinical cardiovascular outcomes. Direct metabolic outcomes such as HOMA-IR, HbA1c, metabolic syndrome, and cardiovascular events were not evaluated. Third, information on dietary intake, physical activity, and uric acid-lowering medications was not available, which may have led to residual confounding or misclassification. In addition, the subgroup analyses according to weight gain status were limited by the small sample size and should be regarded as hypothesis-generating. Fourth, the study population consisted of non-obese working women, which may limit generalizability to men, obese populations, or non-working populations. Fifth, weight gain since early adulthood was assessed by self-report and may be subject to recall bias. Finally, although fasting blood samples were used, the data were derived from routine occupational health examinations, and other unmeasured differences in examination programs across facilities may have influenced the findings.

## Conclusions

In this exploratory cross-sectional study of non-obese working women, serum uric acid was not independently associated with the TG/HDL-C ratio after adjustment for confounding factors. These findings suggest that serum uric acid may reflect underlying metabolic status, but they do not indicate a direct association with insulin resistance or clinical cardiovascular outcomes. Because the TG/HDL-C ratio is a surrogate marker, the results should be interpreted cautiously and regarded as hypothesis-generating. In occupational health settings, these results may support considering serum uric acid in conjunction with BMI and weight change rather than in isolation. Further longitudinal studies are needed to clarify the clinical significance of these findings.

## Electronic supplementary material

Below is the link to the electronic supplementary material.

Supplementary Material 1

## Data Availability

The datasets analyzed during the current study are not publicly available due to confidentiality restrictions related to occupational health data but are available from the corresponding author on reasonable request, subject to applicable ethical and legal restrictions.
