# Supplementary material for: Serum uric acid and TG/HDL-C ratio in non-obese working women: an exploratory cross-sectional study
Source: BMC Endocr Disord. 2026 Jun 27;26:279. doi: 10.1186/s12902-026-02380-1 (PMC13591836; doi:10.1186/s12902-026-02380-1)
Supplement: Supplementary file 1 [file 12902_2026_2380_MOESM1_ESM.docx]

Supplementary Table 1. Variables derived from health checkup questionnaires

| Variable | Description | Response format |
| --- | --- | --- |
| Current smoking status | Current smoking | Yes / No |
| Alcohol use | Regular alcohol consumption | Yes / No |
| Weight gain | ≥10 kg weight gain since age 20 | Yes / No |
| Hypertension medication | Use of antihypertensive medications | Yes / No |
| Diabetes medication | Use of antidiabetic medications | Yes / No |

These variables were derived from questionnaire data collected as part of routine health checkups across multiple facilities. Because the original questionnaires varied by facility, responses were harmonized into binary variables for analysis.

Supplementary Table 2. Multivariable regression analysis of the association between serum uric acid and the TG/HDL-C ratio stratified by weight gain status.

| Subgroup | n | β | 95% CI | P value |
| --- | --- | --- | --- | --- |
| No weight gain (<10 kg) | 561 | 0.032 | -0.012 to 0.077 | 0.151 |
| Weight gain (≥10 kg) | 68 | -0.152 | -0.406 to 0.103 | 0.238 |

Values are presented as regression coefficients with 95% confidence intervals. Models were adjusted for age, BMI, systolic blood pressure, antihypertensive medication use, antidiabetic medication use, smoking status, and alcohol consumption. One participant with missing information on weight gain status was excluded from the subgroup analysis. BMI, body mass index; CI, confidence interval; TG/HDL-C, triglyceride-to-high-density lipoprotein cholesterol ratio.
